# Intrachromosomal Rearrangements in Rodents from the Perspective of Comparative Region-Specific Painting

**DOI:** 10.3390/genes8090215

**Published:** 2017-08-30

**Authors:** Svetlana A. Romanenko, Natalya A. Serdyukova, Polina L. Perelman, Svetlana V. Pavlova, Nina S. Bulatova, Feodor N. Golenishchev, Roscoe Stanyon, Alexander S. Graphodatsky

**Affiliations:** 1 Institute of Molecular and Cellular Biology, Siberian Branch of the Russian Academy of Sciences, 630090 Novosibirsk, Russia; ns3032@yandex.ru (N.A.S.); polina.perelman@gmail.com (P.L.P.); graf@mcb.nsc.ru (A.S.G.); 2 Synthetic Biological Unit, Novosibirsk State University, 630090 Novosibirsk, Russia; 3 A.N. Severtsov Institute of Ecology and Evolution, Russian Academy of Sciences, 119071 Moscow, Russia; swpavlova@mail.ru (S.V.P.); bulatova.nina@gmail.com (N.S.B.); 4 Zoological Institute, Russian Academy of Sciences, 199034 Saint-Petersburg, Russia; f_gol@mail.ru; 5 Department of Biology, Anthropology Laboratories, University of Florence, 50122 Florence, Italy; roscoe.stanyon@unifi.it

**Keywords:** centromere shift, chromosome painting, comparative cytogenetics, fluorescence in situ hybridization, inversion, microdissection, small mammals, voles

## Abstract

It has long been hypothesized that chromosomal rearrangements play a central role in different evolutionary processes, particularly in speciation and adaptation. Interchromosomal rearrangements have been extensively mapped using chromosome painting. However, intrachromosomal rearrangements have only been described using molecular cytogenetics in a limited number of mammals, including a few rodent species. This situation is unfortunate because intrachromosomal rearrangements are more abundant than interchromosomal rearrangements and probably contain essential phylogenomic information. Significant progress in the detection of intrachromosomal rearrangement is now possible, due to recent advances in molecular biology and bioinformatics. We investigated the level of intrachromosomal rearrangement in the Arvicolinae subfamily, a species-rich taxon characterized by very high rate of karyotype evolution. We made a set of region specific probes by microdissection for a single syntenic region represented by the p-arm of chromosome 1 of *Alexandromys oeconomus*, and hybridized the probes onto the chromosomes of four arvicolines (*Microtus agrestis*, *Microtus arvalis*, *Myodes rutilus*, and *Dicrostonyx torquatus*). These experiments allowed us to show the intrachromosomal rearrangements in the subfamily at a significantly higher level of resolution than previously described. We found a number of paracentric inversions in the karyotypes of *M. agrestis* and *M. rutilus*, as well as multiple inversions and a centromere shift in the karyotype of *M. arvalis*. We propose that during karyotype evolution, arvicolines underwent a significant number of complex intrachromosomal rearrangements that were not previously detected.

## 1. Introduction

Classical chromosome staining and banding allows some appreciation of the extent of chromosomal evolution across animal species. Molecular cytogenetics puts karyological comparisons onto a more secure footing. Chromosome painting has allowed researchers to access, with a high degree of confidence, interchromosomal rearrangements that differentiate mammalian karyotypes over evolutionary time. Well over 100 species of mammals were studied with chromosomal painting. The method has mapped evolutionary conserved syntenic segments, fusions, and fissions over a wide phylogenetic array of species. These data were sufficient to make reasonable hypotheses on a fundamental goal of comparative cytogenetics—the reconstruction of ancestral karyotypes at principal phylogenetic nodes on the placental mammalian tree.

The advent of whole-genomic sequencing has provided new tools for the analysis of genome-scale data and comparison of species genomes at the level of DNA sequence. Bioinformatics led to a series of attempts to reconstruct the architecture of the ancestral eutherian karyotype [1,2,3]. Although these early sequence level reconstructions of the ancestral genome of placental mammals supported most chromosome painting results, there was also a significant number of differences. It is necessary to carefully evaluate why these methods often yielded different results.

Both approaches reconstruct evolutionary genomic changes by identifying the most parsimonious number of rearrangements of ancestral building blocks, albeit on vastly different scales. As expected, bioinformatics revealed more conserved segments and a higher number of syntenic associations then cytogenetics. However, it is important to note that the differences did not correlate with increased resolution provided by DNA sequence comparisons [3]. The discrepancy between cytogenetic and bioinformatic models of the ancestral genome are better explained by the limited taxon sampling and/or algorithms in bioinformatic analysis that do not take into account evolutionary rate variation among lineages [3]. This conclusion is supported by the fact that over time, as more genome assemblies and better algorithms became available, the bioinformatics and cytogenetic views of the ancestral genome converged [4]. However, there still remain significant differences.

The main weakness of the most commonly used molecular cytogenetics method, chromosome painting, is that intrachromosomal rearrangements go undetected [5]. At the molecular cytogenetic level, intrachromosomal rearrangements can be identified by hybridizing cloned DNA such as bacterial artificial chromosomes (BACs) or probes specific for particular chromosome regions, such as those derived from microdissection. However, up to now, these methods were only used in a limited number of mammalian taxa: marsupials, Primates, Carnivora, Rodentia, Chiroptera, Perissodactyla (see Table 1 in [6,7,8]).

Probes obtained by microdissection were first applied to, and mainly used for clinical analysis, but they have also proved to be useful tools for comparative genomics. Microdissection-derived regional chromosome probes were efficiently used to determine the orientation of conserved blocks within a chromosome, the order of subchromosomal segments within large syntenic blocks, and were especially helpful in identifying intrachromosomal rearrangements [9,10].

The use of cloned DNA probes in fluorescence in situ hybridization (FISH) such as BACs not only permitted high-resolution investigations, but efficiently revealed intrachromosmomal rearrangements such as inversions at even a higher level of resolution. Results of BAC-FISH investigations of primate genomes led to the discovery of a new type of rearrangement—centromere repositioning or shift [11], that is, the movement of the centromere without a change in marker order (i.e., without inversions). It is notable that this phenomenon is very arduous to track, and was not discovered at the DNA sequence level. Later it was shown that this type of rearrangement is not rare, and centromere shifts have frequently led to the evolutionary emergence of new centromeres in many different groups of mammals [12,13,14,15].

Arvicolinae is a subfamily of more than 150 species of rodents characterized by great karyotypic variability. This variability was generated over a relatively short period of evolutionary time—less than 10 million years [16,17]. Arvicolinae is a taxonomically complex and debated group of rodents. Some researchers recognize 26 genera divided into ten tribes but with two genera of unknown positions [18]. A more recent publication taking Russian fauna into consideration recognized 30–32 genera, grouped into 10–11 tribes [19].

The entire subfamily represents a useful model to study chromosomal evolution. Comparative cytogenetic investigations have shown that many fusion/fission rearrangements occurred in the evolution of the subfamily [20,21,22,23]. Centromeric shifts were also suggested as a mechanism that helped differentiate the karyotypes of common voles of the *Microtus arvalis* group [24]. It was sometimes proposed that inversions, with variable contributions in different phylogenetic lineages, have probably played an important role in the karyotype evolution of Arvicolinae [20,21,23].

Here, we focused on the evolutionary conserved syntenic block 7 of the ancestral Arvicolinae karyotype (AAK, [25]). The region is homologous to the distal part of chromosome 1 of *Microtus agrestis* (MAGR) and is present as a separate chromosome in the majority of vole species studied and in some other arvicolines [20,21,22,25]. Although this chromosome varies little in G-banding, the position of the centromere varies considerably. The different morphologies of this conserved syntenic block suggest that it has been altered by centromere shifts, as well as other types of intrachromosomal rearrangements [20]. We generated a set of region-specific probes from the p-arm of *Alexandromys oeconomus* (tribe Arvicolini) chromosome 1 (AOEC1 = AAK7) by microdissection. For analysis, we chose *Myodes rutilus* (MRUT) and *Dicrostonyx torquatus* (DTOR) representing tribes Myodini and Dicrostonychini in Arvicolinae, and *Microtus agrestis* and *Microtus arvalis* (MARV), belonging to the Arvicolini. Comparative chromosome painting of this set of probes in four representative arvicoline species uncovered a number of cryptic intrachromosomal rearrangements, and provides an explanation for changes in centromere position.

## 2. Materials and Methods

### 2.1. Ethics Approval

All experiments were approved by the Ethics Committee on Animal Experiments of the Institute of Molecular and Cellular Biology, Siberian Branch of the Russian Academy of Sciences, Russia (approval No. 31 of August 6, 2015).

### 2.2. Species Sampled

*M. agrestis*, *M. arvalis*, *A. oeconomus*, *M. rutilus*, and *D. torquatus* cell lines were retrieved from the IMCB SB RAS cell bank (“The general collection of cell cultures”, No. 0310-2016-0002). The origin of each sample, establishment of cell lines, and karyotype description for each species studied were previously reported [20,22,26].

### 2.3. Chromosome Preparation and Chromosome Staining

Chromosome suspensions were obtained from cell lines according to earlier published protocols [27,28]. G-banding was performed on chromosomes of all species prior to FISH, using the standard trypsin/Giemsa treatment procedure [29].

### 2.4. Microdissection, Probe Amplification and Labeling

Microdissection of the p-arm of the *A. oeconomus* chromosome 1 was performed on G-banded chromosomes as described in [30]. Ten copies of each region were collected. Chromosomal DNA was amplified and labeled using WGA kits (Sigma-Aldrich, Saint Louis, MO, USA). In total, we obtained five region-specific painting probes covering the whole p-arm of the AOEC chromosome 1.

### 2.5. Fluorescence in situ Hybridization

The painting probes were labeled with either biotin or digoxigenin by degenerate oligonucleotide-primed polymerase chain reaction amplification as described previously [22,26,31,32]. We used dual-color FISH with different pairwise combinations of probes to establish their relative localization. FISH was performed according to previously published protocols [33,34]. Images were captured using VideoTest-FISH software (Zenit, Saint-Petersburg, Russia) with a JenOptic charge-coupled device (CCD) camera (Jena, Germany) mounted on an Olympus BX53 microscope (Shinjuku, Japan). Hybridization signals were assigned to specific chromosome regions defined by G-banding pattern captured by the CCD camera prior to FISH. All images were processed using Corel Paint Shop Pro X2 (Corel, Ottawa, ON, Canada).

## 3. Results

**Figure 1:**
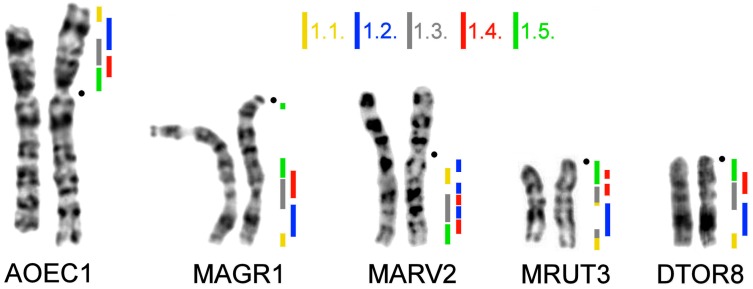
Localization of *A. oeconomus* region-specific probes onto chromosomes of members of the Arvicolinae. AOEC—*A. oeconomus*, MAGR—*M. agrestis*; MARV—*M. arvalis*; DTOR—*D. torquatus*. Colored lines and numerals correspond to individual microdissection-derived painting probes.

We made a set of region-specific painting probes of the p-arm of chromosome 1 of *A. oeconomus* (Arvicolini tribe). FISH was performed on metaphase chromosomes of selected species to define the precise localization of each probe. In total, we obtained five partly overlapping probes covering the whole p-arm of AOEC1 = AAK7 (Figure 1). Based on comparative chromosomal studies, the size of AOEC1p approximately corresponded to mouse chromosome 9 [26]. According to the assembly of the mouse genome GRCm38/mm10, the size of the chromosome is 124 Mbp, so a rough estimate of each microdissected probe size was about 25 Mb.

**Figure 2:**
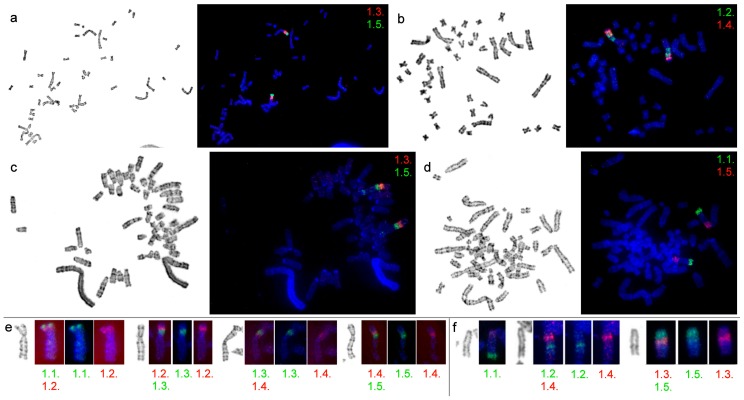
FISH of microdissection-derived painting probes on chromosomes of different species: (**a**) *M. arvalis*; (**b**) *M. arvalis*; (**c**) *M. agrestis*; (**d**) *D. torquatus*; (**e**) *A. oeconomus*; (**f**) *D. torquatus.* G-banded chromosomes are shown on the left, the image with localization of both probes—on the right. Separate images for the green and red signals are presented (**e**,**f**). Color-coded number of the probe is shown for each image.

The set of probes was used for the comparison of the chromosomes of four species: *M. agrestis* and *M. arvalis* (both from the Arvicolini tribe), *M. rutilus* (Myodini), and *D. torquatus* (Dicrostonychini). In all cases, the probes produced clear and bright signals sufficient for comparative investigation. Examples of fluorescence in situ hybridizations are shown in Figure 2.

The set of probes marked chromosome 8 of *D. torquatus*. The pattern of the probe distribution was the same as in the karyotype of *A. oeconomus* (Figure 2). A similar localization of the probes was seen in the distal part of *M. agrestis* chromosome 1. However, probe 1.5. produced a small signal in the pericentromeric region of the chromosome of *M. agrestis* (Figure 2).

**Figure 3:**
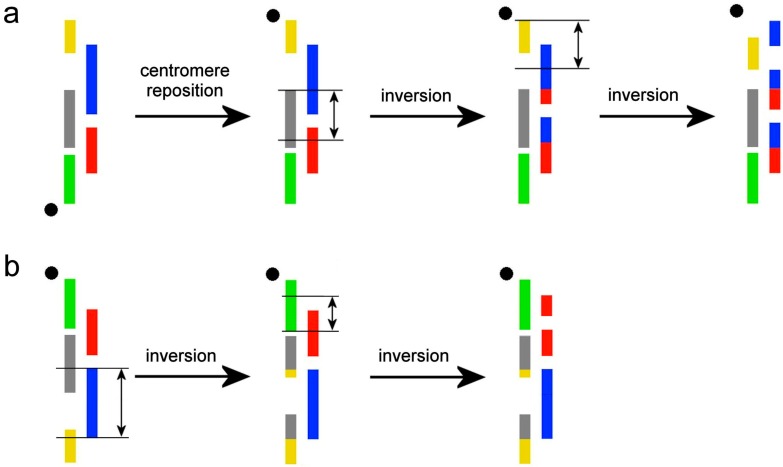
A putative scheme for intrachromosomal rearrangements (**a**) in the q-arm of *M. arvalis* chromosome 2; (**b**) in *M. rutilus* chromosome 3. The black circles mark the position of the centromere. The black lines and vertical arrows mark the regions of inversions. The colors of the microdissection-derived probes correspond to those on Figure 1.

Five probes hybridized the q-arm of chromosome 2 in the karyotype of *M. arvalis* and demonstrated the presence of intrachromosomal rearrangements (Figure 3a). In the karyotype of *M. rutilus*, probes 1.1., 1.3., and 1.4. each produced two signals on chromosome 3 (Figure 3b).

In the karyotypes of *D. torquatus*, *M. agrestis*, and *A. oeconomus*, we detected a similar hybridization pattern by the probes (Figure 1). We suggest that the probe order found in *D. torquatus*, *M. agrestis*, and *A. oeconomus* could be considered as ancestral for at least the *Microtus* genus. Probe 1.5. produced small signals in the pericentromeric region of chromosome 1 of *M. agrestis* (Figure 2) which may have been due to repeat sequences. This probe was derived from the pericentromeric region of AOEC1, and could include some repeated sequences that had high homology to the pericentromeric region sequences of various arvicolines.

The localization of the five probes on *M. arvalis* chromosome 2 is best interpreted as due to intrachromosomal rearrangements. In Figure 3b we illustrated the hypothesized changes that may have led to formation of the hybridization pattern that we found, as compared to the ancestral pattern. We propose that a centromere shift has occurred. Apparently, two paracentric inversions reshuffled syntenic blocks of the *M. arvalis* chromosome 2q. In *M. rutilus*, multiple signals given by probes 1.1., 1.3., and 1.4. indicated the probable presence of two paracentric inversions, which gave origin to the chromosome MRUT3 (Figure 3a).

## 4. Discussion

Up to now, intrachromosomal rearrangements were identified by molecular cytogenetic methods in only a limited number of species. Whole chromosome-specific probes were mostly used to document interchromosomal rearrangements (translocations). Occasionally, chromosome paints also reveal intrachromosomal rearrangements such as inversions, but this is the exception, not the rule [23,35,36,37]. Most intrachromosomal rearrangements were identified according to the localization of different types of region-specific probes such as cloned DNA, microdissected probes, and bioinformatic approaches.

In mammals, the most thoroughly studied intrachromosomal rearrangements are those in primates, but data are available for some hoofed mammals, for which fine-scale comparative genomic data are available [38]. For example, the application of region-specific painting and BAC probes delineated the orientation of evolutionarily conserved segments with respect to centromere positions in Equidae [9,15,39]. FISH cohybridization experiments with BAC clones and bioinformatic methods clarified the mechanisms of karyotype evolution in the taxon, showing that the centromere indeed changed its position during evolution [40,41,42]. Extensive investigations of primates allowed researchers to identify a significant number of cryptic intrachromosomal rearrangements differentiating the karyotypes of monkeys and humans [6,43,44,45]. The mapping of mouse complementary DNA clones of X-linked genes showed that the X chromosomes of two species of Ryukyu spiny rat differed by centromere repositioning [46].

Microdissected derived probes showed that intrachromosomal rearrangements distinguished the X chromosomes of two African antelope species [47]. Investigations using microdissected probes also showed that the X chromosome of five *Microtus* species differed due to intrachromosomal rearrangements [48]. Cross-species comparative multicolor banding with probes obtained from mouse chromosomes allowed cytogeneticists to detect inversions and evolutionary new centromeres in nine muroid species [7]. It is important to note that in some cases, intrachromosomal rearrangements have provided important phylogenetic information, as for example in Bovidae [47,49].

The Arvicolinae present one of the best models to investigate intrachromosomal rearrangements. First of all, molecular data have recently resolved the main phylogenetic branches and eliminated long standing taxonomic problems in the subfamily [16,17,50]. Secondly, there are karyotypes for almost all species, conserved segments have been identified, and an ancestral karyotype of Arvicolinae has been reconstructed [20,22,25]. Finally, the presence of sibling species (morphologically similar, but with distinct karyotypes) makes cytogenetic data particularly important for the identification of some arvicoline species, particularly in voles [51]. However, cladistic analysis of chromosomal characters, in spite of the great karyotype variability between species, has not yet resolved phylogenomic relationships between some species. This lack of resolution was probably due to the predominance of Robertsonian rearrangements, which are prone to convergence [20,22]. To resolve this problem, it was proposed that documenting intrachromosomal rearrangements within large conserved syntenic blocks might help to resolve at least some of these complex relationships [20]. Further, the identification and description of rearrangements inside conserved segments may well contribute to a better understanding of the mechanisms of karyotype evolution.

AAK7 is a large ancestral segment that was preserved intact in the genomes of all arvicoline studied, and has similar G-banding patterns, but different centromere positions [20,25]. To identify rearrangements in the conserved syntenic block, we generated a set of region-specific probes from the p-arm of *A. oeconomus* chromosome 1 (AOEC1 = AAK7) by microdissection. This species was selected because its low chromosome number allows easy chromosome identification on a metaphase plate for microdissection. For analysis, we chose four species representing different arvicoline genera and tribes: (1) *D. torquatus* (tribe Dicrostonychini), (2) *M. rutilus* belonging to tribe Myodini, (3) *M. agrestis*, and (4) *M. arvalis*, which both belong to the *Microtus* subgenus inside the Arvicolini tribe. Genus *Dicrostonyx* is one of the most basal arvicolines, it diverged about 6.4 million years ago (MYA). The tribe Myodini diverged about 3.2. MYA. Probes were obtained from *A. oeconomus*, which belong to the *Alexandromys* subgenus (1.9 MYA) of the Arvicolini tribe and diverged earlier than the *Microtus* subgenus (1.4 MYA) which is part of the same tribe [16,17,50].

The considerable number of intrachromosomal rearrangements that were detected in the karyotypes of these four species supports the hypothesis that inversions and centromere shifts are frequent in the karyotype evolution of animals. The results also support the conclusion that the frequency and importance of intrachromosomal rearrangements is often significantly underestimated, especially in some groups of species [52,53,54].

## 5. Conclusions

Previously, it was proposed that intrachromosomal rearrangements may be up to four times more frequent than other chromosomal rearrangements; however, chromosome painting—the most commonly used molecular cytogenetic method—leaves almost all inversions undetected [20,55]. The application of region-specific probes has, up to now, been limited. Here, we investigated the so-called evolutionarily conserved syntenic autosomal elements in Arvicolinae by using region-specific paints. We were able to document numerous intrachromosomal rearrangements, and found that chromosomes have been subject to significant reshuffling in at least two of the four species studied. It is clear that inversions and centromere repositioning in mammalian species still remains poorly documented. A reliable evaluation of the importance of these types of rearrangements for karyotype evolution and their utility for phylogenomics will require further investigations involving a broader array of species, and wider application of region-specific probes.

Unfortunately, it is not yet possible to make broad bioinformatic comparisons of arvicoline genomes because of limited whole-genome sequencing data for the taxon. However, we need to stress that in some cases, it is very difficult to identify intrachromosomal rearrangements, even with the use of bioinformatics. This requires not only the data from full genome sequencing, but also a high quality and full chromosome assembly based on physical or optical mapping. Assemblies based on a reference genome are not sufficient. Furthermore, detection and investigation of intrachromosomal rearrangements in Arvicolinae with microdissection-derived or other region-specific probes will be useful not only for resolving complex phylogenetic relationships, but also for uncovering the mechanisms of chromosome evolution, and for the clarification of the role of chromosome rearrangements in the speciation of this spectacularly diverse taxon.
